# Filling the Gaps: Current Research Directions for a Rational Use of Probiotics in Preterm Infants

**DOI:** 10.3390/nu10101472

**Published:** 2018-10-10

**Authors:** Arianna Aceti, Isadora Beghetti, Luca Maggio, Silvia Martini, Giacomo Faldella, Luigi Corvaglia

**Affiliations:** 1 Neonatal Intensive Care Unit, AOU Bologna, Department of Medical and Surgical Sciences (DIMEC), University of Bologna, 40138 Bologna, Italy; i.beghetti@gmail.com (I.B.); silvia.martini4@gmail.com (S.M.); giacomo.faldella@unibo.it (G.F.); luigi.corvaglia@unibo.it (L.C.); 2 Department of Woman and Child Health, Obstetric and Neonatology Area, Fondazione Policlinico Universitario A. Gemelli IRCCS, 00168 Rome, Italy; luca.maggio@fastwebnet.it

**Keywords:** preterm infant, probiotic, human milk, probiotic strain, safety

## Abstract

The use of probiotics among very low-birth-weight infants is constantly increasing, as probiotics are believed to reduce the incidence of severe diseases such as necrotizing enterocolitis and late-onset sepsis and to improve feeding tolerance. However, despite the enthusiasm towards these products in neonatal medicine, theoretical knowledge and clinical applications still need to be improved. The purpose of this review is to give an overview of the most important gaps in the current literature about potential uses of probiotics in preterm infants, highlighting promising directions for future research. Specifically, further well-designed studies should aim at clarifying the impact of the type of feeding (mother’s milk, donor milk, and formula) on the relationship between probiotic supplementation and clinical outcome. Moreover, future research is needed to provide solid evidence about the potential greater efficacy of multi-strain probiotics compared to single-strain products. Safety issues should also be addressed properly, by exploring the potential of paraprobiotics and risks connected to antibiotic resistance in preterm infants. Last, in light of increasing commercial and public interests, the long-term effect of routine consumption of probiotics in such a vulnerable population should be also evaluated.

## 1. Introduction

Despite continuous improvements in neonatal medicine, prematurity represents the leading cause of both neonatal and childhood mortality through age five years worldwide [1]. Diseases such as necrotizing enterocolitis (NEC) and late-onset sepsis (LOS) pose a serious threat for preterm infants, and are included among the leading causes of morbidity and mortality in this population [2].

There is a growing body of literature directed towards the implementation of preventive measures which could reduce the incidence and severity of these diseases, possibly leading to an improvement in neonatal short- and long-term outcomes [3]. Among these interventions, probiotics appear to be very promising; they have been recently described as living “a golden age” in neonatal medicine [4].

Despite such enthusiasm, it is still difficult to make precise recommendations on the use of probiotics in preterm infants. Recent systematic reviews and meta-analyses of the literature have suggested a beneficial role of probiotics for preventing NEC [5,6,7] and LOS [8,9], and for improving feeding tolerance by shortening the time to achieve full enteral feeding (FEF) [10,11]; furthermore, it has been suggested that probiotic administration might lead to a global improvement in neonatal health by reducing mortality rates and length of hospital stay [12,13,14]. However, most of the studies included in these systematic reviews and meta-analyses have not properly addressed all of the potential confounding factors which might interfere in shaping the relationship between probiotic administration and clinical outcomes, thus failing to provide to the clinician a meaningful answer as to which probiotic strain should be used, how long probiotic supplementation should be continued, and which group(s) of infants would likely benefit from the intervention.

Despite concerns about the efficacy and, more importantly, the safety of available probiotic products [15], a recent survey of US Neonatal Intensive Care Units (NICUs) participating to the Vermont Oxford Network has shown that probiotic use among very low-birth-weight (VLBW) infants is increasing, and that there is a wide variability in their indications and in the probiotic products themselves [16].

The purpose of this review is to give an overview of the most important gaps in the current literature about potential uses of probiotics in preterm infants, highlighting promising directions for future research.

## 2. Defining the Target: Gut Microbiota vs. Clinical Outcomes

In infants, as well as in children and adults, the rationale for using probiotics to treat or prevent any disease relies on their potential ability to restore a healthy gut microbiota. Probiotic action in the gut is mediated through several mechanisms, which include the up- and down-regulation of genes involved in inflammation and cytoprotection, improvement of gut barrier function, production of metabolites such as short-chain fatty acids which modulate the growth of pathogenic bacteria, and competition with other microorganisms [17].

When talking about preterm infants, a clear definition of this probiotic target is challenging, as the characterization of a healthy gut microbiota in these infants is not univocal, being influenced by several factors such as place of birth, mode of delivery, feeding characteristics, antibiotic use, and NICU environment [18]. In addition, despite a generally clear association between dysbiosis and disease, the causality and reversal of disease in response to probiotic-induced changes in gut microbiota have not yet been demonstrated [19]. For this reason, the target for probiotic intervention in preterm infants at present cannot be exclusively microbiological (in other words, a gut microbiota with distinctive “healthy” features), but still needs to be clinical.

Given this premise, a clear definition of clinical outcomes whose improvement might be related to probiotic administration is fundamental. In most clinical trials, sample size calculation is based on single pre-specified outcomes (i.e., mortality, NEC, LOS, feeding tolerance), but other outcomes, for which these studies are probably underpowered, are often analyzed and their results are included among relevant conclusions. This constitutes a limitation of these studies’ conclusions, which hopefully should be overcome in the future by calculating the sample size on multiple outcomes.

## 3. Exploring Feeding Contribution: Human Milk vs. Formula

Infant nutrition in early life has been recognized to play a key role in shaping future health [20]. Specifically, human milk (HM) is known to exert a series of beneficial effects for the infant, including improved neurological, immunological, and metabolic outcomes [21]. In this respect, exclusive HM-feeding appears to be even more important for preterm infants, especially for those born with a VLBW [22]. The beneficial effects of HM are related to its peculiar nutritional and functional components, such as long-chain polyunsaturated fatty acids, immunomodulatory proteins (i.e., lactoferrin, immunoglobulin), and HM oligosaccharides (HMOs) [23]. Some of these HM components have been also related to the decrease in NEC incidence and severity documented in infants receiving exclusive HM [24]. The unique composition of HM possibly confers protection against NEC through several mechanisms implicated in the pathogenesis of the disease, such as reduction in gastric pH, improvement in intestinal motility, and decrease in epithelial permeability. In addition, immunoglobulin A and lactoferrin, contained in HM, might modulate gut inflammation, and HMOs are thought to prevent bacterial adhesion to the intestinal mucosa and exert a prebiotic action on gut microbiota [25]. Furthermore, it is well recognized that HM itself harbors a specific microbiota, which contributes to drive the establishment of the infant gut bacterial community [26]. In this regard, features of gut microbiota in breastfed vs. formula-fed infants are quite different: breastfed infants have a higher abundance of *Bifidobacterium* spp., *Staphylococcus* spp., and *Streptococcus* spp., while *Bacteroides* spp., *Clostridium* spp., *Enterobacteriaceae*, and *Lachnospiraceae* dominate in formula-fed infants [27].

A recent systematic review examined the effect of different enteral feeding strategies, including feeding type and probiotic supplementation, on the establishment of gut microbiota in preterm infants [28], with the aim of identifying feeding factors which would promote gut colonization with beneficial bacteria. Both mother’s own milk (MOM) and probiotics were found to promote a healthy gut microbiota, by increasing microbial diversity and promoting colonization with *Bifidobacteria*.

Since the effects of both HM and probiotics on gut microbiota and clinical outcomes such as NEC and LOS have been advocated in so many clinical trials and literature reviews, it is quite surprising that the two interventions have not been examined together. It is plausible that nutrition and supplemental probiotics could act together in the preterm infant’s gut, leading to a differential effect of exogenous bacteria depending on type of feeding. Despite the theoretical likeliness of this hypothesis, type of feeding is rarely detailed or considered among confounding factors in trials about probiotics. One might speculate that probiotics would act preferentially in formula-fed infants, as they would counterbalance the negative effect of formula feeding on gut microbiota. However, unexpectedly even for the authors themselves, one clinical trial reported that a combination of probiotic strains (*Lactobacillus acidophilus* and *Bifidobacterium bifidum*) was effective on NEC only in VLBW infants who were exclusively breastfed, but not in those receiving formula [29]. Similarly, two meta-analyses of randomized controlled trials (RCTs) documented a reduction in the incidence of LOS and in the time to achieve FEF only in HM-fed preterm infants [9,11]. More recently, the effect of probiotics on NEC was found to be more pronounced in cohorts where higher proportions of neonates were exclusively breastfed [30].

The available literature does not offer a clear justification for these findings: one hypothesis is that VLBW infants exposed to formula might have a higher baseline risk for diseases such as NEC and feeding intolerance, because intact bovine proteins in infant formula can contribute to intestinal inflammation [25]. Furthermore, it is plausible that the effect of probiotics on clinical outcomes could be mediated by functional HM components such as prebiotic HMOs, immunological factors, and probiotic bacteria [31]. In this respect, recent data suggest that a low abundance of a specific HMO in MOM (disialyllacto-N-tetraose, DSLNT) could represent a marker of the susceptibility of preterm infants to NEC [32], thus highlighting the fundamental role of the crosstalk between HM prebiotic components, gut and HM microbiota, and probiotics in the development of the disease.

While the role of MOM in the prevention of NEC has been established, less is known about donor HM (DHM). In a multicenter clinical trial, the availability of DHM was associated with a 10% increase in MOM availability and a 2.6% decrease in the rate of NEC [33]. Similarly, the introduction of DHM and probiotic supplementation in two clinical settings was linked to a significant reduction in neonatal mortality and to a non-significant decrease in NEC rates [34]. On the contrary, the use of DHM as a supplement to MOM was not associated with any significant benefit in terms in mortality and morbidity indexes, nor in neurodevelopment at 18 months corrected age [35].

The interaction between DHM and probiotic supplementation in shaping neonatal clinical outcomes in far from clear. The manipulation process of DHM, which includes several steps such as freezing, thawing, pooling, and pasteurization, is likely to have a major impact on HM functional components [36]. Whether the changes in DHM composition induced by its manipulation translate into a clinically relevant effect is yet to be demonstrated. Recently, the effect of different feeding types, including MOM, DHM, and formula, on the features of gut microbiota in preterm infants was analyzed: compared to DHM-fed infants, gut microbiota in MOM-fed infants was richer in Bifidobacteriaceae and poorer in Staphylococcaceae, Clostridiaceae, and Pasteurellaceae. Despite these differences, gut microbiota profiles in MOM-fed and DHM-fed infants were more similar than those observed in formula-fed infants [37]. The clinical significance of these observations deserves to be assessed in further trials.

In summary, given the differential effect of MOM, DHM, and formula on both gut microbiota and neonatal clinical outcomes, it is fundamental that further studies exploring probiotic supplementation in preterm infants will plan to describe in greater detail and adjust their data for feeding characteristics of included infants, in order to limit potential confounding factors (i.e., percentage of MOM, duration of exclusive MOM, characteristics of HM fortification, etc.). Even better, it would be ideal to restrict inclusion criteria to infants fed homogeneously (MOM, DHM, or formula), thus overcoming the impracticability of randomizing preterm infants according to type of feeding.

## 4. Choosing the Probiotic: Is There Strength in (Strain) Numbers?

The strain-specificity of the probiotic effect has been accepted for decades as a pivotal principle of probiotic science and regulation [38]. More than 20 years from the first RCT showing that probiotics could be effective in reducing NEC, several RCTs and cohort studies reporting on NEC, LOS, and/or mortality in preterm infants receiving probiotic supplementation have been conducted. Multiple different probiotic strains or combinations have been used and some of them have been shown to exert beneficial effects. However, it has not been clarified whether a single probiotic or a mixture of probiotics would be more effective in improving preterm infants’ outcome, and which probiotic strain or mixture should be better used. Numerous meta-analyses summarizing currently available data have recently been published [4,6,9,11,13,14,30,39,40,41,42]: data from these meta-analyses suggest that, beyond the overall benefit, a combination of different strains or species may have advantages over single probiotic organisms [6,9,14,30,39]. Since the same probiotic strain was used in very few studies, strain-specific sub-meta-analyses are difficult to perform, and strain-specific meta-analyses have been conducted only twice [40,41]. In order to provide an overview on the use of probiotics in preterm infants at a strain level and to identify strains with the greatest efficacy, van den Akker et al. carried out a network meta-analysis, combining evidence from direct and indirect comparison across several competing interventions [7]. Single- or multi-strain studies reporting on NEC, LOS, time to FEF, or overall mortality were included. Only a minority of the studied interventions, both single-strain and multi-strain/multi-species, was found to be effective. There was no clear overlap of certain strains which were significantly effective on multiple outcome domains. Most strains or combinations of strains only showed trends towards efficacy, whereas other strains did not demonstrate any. The authors suggested that a lack of effect might be either due to understudied species or to a true lack of effect of certain strains.

The extreme variety of probiotics used in neonatology might lead to conflicting conclusions. The variability of the strains and protocols in the trials included in various meta-analyses is often presented as a weakness. However, given the consistently decreased risk of NEC in RCTs using different probiotic regimens, it could be argued that most of the investigated interventions improve the health of the participants beyond any placebo effect. This evidence might indicate that the concept of strain-specific effects of probiotics may not be relevant to certain outcomes such as NEC [43]. Because of the complexity of the normal gut microbiome and of various cascades involved in the pathogenesis of NEC, different strains may exert their beneficial effect by different pathways. Thus, some authors suggested that commonly used probiotic strains share core benefits providing ‘non-specific’ or ‘generic’ protection [44,45,46]. While some specific mechanisms are rare and present in only a few strains of commonly studied probiotic species, some mechanisms might be frequently observed at a species and even a genus level [45,47]. Shared mechanisms exist among taxonomic groups that include many different strains [48]. On these bases, a multi-strain or multi-species probiotic [49] could be more effective than a single-strain product: more strains give more chances of success for a beneficial effect, provide a greater microbiota diversity and thus more potential niches in combination with individually determined host factors, allow a broader efficacy spectrum, and may exert additive or synergistic effects. However, a combination of probiotic strains does not necessarily ensure, in itself, a greater efficacy than a single-strain probiotic, as different probiotic strains may exert mutual inhibitory properties through the production of antimicrobial substances or differential gene expression [50,51].

Further structured research is needed to address the benefits of single-strain versus multi-strain or multi-species probiotic products in preterm infants. Head-to-head human intervention studies should be performed comparing a multi-strain product with its single-strain components and a placebo. Furthermore, in vitro and animal studies should aim to identify safe probiotic combinations that show additive or synergistic properties, avoiding potential antagonistic effects.

## 5. Addressing Safety Concerns: Time for Ghost Probiotics?

The routine administration of probiotics to preterm infants is hindered by concerns about the safety of commercially available products. Reasons of concern include fear of probiotic-related sepsis, transmission of antibiotic resistance, and non-availability of high-quality products.

Although none of the meta-analyses on probiotic supplementation in preterm infants reported any serious adverse events in infants receiving probiotics, there are several reports describing the occurrence of probiotic-related infections such as sepsis, pneumonia, and meningitis [31]. In addition, the genome of probiotic bacteria is known to contain genetic elements which confer resistance to several broad-spectrum antibiotics, such as glycopeptides, aminoglycosides, mono-bactams, and fluoroquinolones [52]; these resistant genes are themselves not harmful, but they can be transferred to other gut bacteria and eventually to opportunistic pathogens, leading to an uncontrolled increase in antibiotic-resistance [53], which might lead to longer-term consequences that outweigh the immediate benefits of probiotic supplementation. Furthermore, in many countries probiotics are considered as food supplements, and thus lack the strict quality regulation of other pharmaceuticals [31]. This is linked to a high risk of product contamination which can have serious consequences, especially when probiotics are administered to vulnerable individuals with weak immune systems, enhanced inflammatory responses, and/or compromised gut barrier, such as preterm infants.

To address the safety issues related to currently available probiotics, research is focusing on inactivated products, the so-called “paraprobiotics”. The concept of paraprobiotics, or ghost probiotics, was developed by Taverniti et al. [54], who defined them as “non-viable microbial cells or crude cell extracts, which, when administered in adequate amounts, confer a benefit on the human or animal consumer”. Inactivation of probiotics has been performed through several methods (i.e., heat, chemical, gamma or ultraviolet rays, sonication), each one having a different effect on cell structure and function [54].

A “probiotic paradox” has been described in relation to paraprobiotics, and is related to the fact that both live and dead microbial cells might exert beneficial responses in the host [55]. Mechanisms of action of paraprobiotics involve the modulation of various steps of the inflammatory and gut immune responses, and theoretical advantages over viable products include enhanced safety and longer shelf-life.

Results of the studies comparing viable vs. non-viable probiotic products on a variety of clinical outcomes have been summarized by Lahtinen et al. The authors concluded that, while it is clear that live bacteria are overall more effective than non-viable ones, there might be some cases where viability is not essential for the health benefit, as suggested by some clinical reports [56]. The evaluation of viable probiotics vs. non-viable ones is further complicated by the fact that, during storage, part of the probiotic population may become “dormant” [57].

At present, few probiotic species, including strains of both *Lactobacillus* and *Bifidobacterium,* have been studied in their inactivated form [58]; current evidence about their potential applications in preterm infants is limited, but it is likely that further research will give new insight into mechanisms of action, most effective and safe probiotic strains to be used in their inactivated form, and most promising clinical applications in preterm infants [59]. 

## 6. Choosing the Study Design: The Challenge of Cross-Colonization

Cross-colonization, or cross-contamination, occurs when a microorganism, including a probiotic, is transferred from one individual or environment to another. Early clinical trials on probiotic supplementation to preterm infants reported some cases of cross-colonization with the probiotic organism. Millar et al. documented transient colonization with *Lactobacillus GG* of one infant, whose twin was receiving probiotic supplementation and was being nursed in an adjacent cot [60]. One RCT investigating the colonization with *Bifidobacterium breve* detected the probiotic bacteria in the feces of 44% of the control infants at six weeks [61]. More recently, Hickey et al. [62] assessed the rate of cross-colonization of specific probiotic organisms within a multicenter RCT [63]. The authors reported a low occurrence of probiotic cross-colonization (7.9%) and environmental contamination, suggesting that this phenomenon may be related to physical proximity to infants receiving probiotics, as well as NICU length of stay. In contrast, the Probiotic in Preterm Infants Study (PiPS), the largest multicenter RCT about probiotics in preterm infants, demonstrated that cross-contamination occurred in 49% of the infants in the placebo arm in all study sites. Furthermore, rates of the primary outcomes did not differ significantly between the probiotic and placebo groups [64]. Nonetheless, further analysis based on the colonization status suggested benefits in the colonized infants irrespective of their allocation to the probiotic or placebo treatment groups [65,66].

Mechanisms of cross-contamination in the NICU have not been satisfactorily explored. They likely include transmission through the contamination of NICU surfaces and the body/hands of caregivers [67]. Even if cross-colonization may not result in ingestion of the probiotic bacteria at beneficial doses in the control group, it may be enough to alter the outcomes. The challenges of cross-contamination include both the potential impact on NICU infants and limitations in assessing the true effect of probiotic supplementation in clinical trials. In order to avoid infant-to-infant probiotic dissemination, Totsu et al. [68] performed a cluster RCT involving 19 NICUs, reporting benefits of single-strain *Bifidobacterium bifidum OLB6378* supplementation in preterm infants. Taking these findings together, cross-colonization should be considered when designing further RCTs. Cluster or cross-over cluster randomized trials in which the NICU is randomized rather than the infant should be considered [40,47,64], as well as more standardized hygiene policies and NICU layouts.

## 7. Conclusions

The interaction between probiotics and the preterm host is extremely complex, due to particular intrinsic and environmental factors. Despite the enthusiasm towards probiotics in neonatal medicine, theoretical knowledge and clinical applications still need to be improved. This review highlights the need for further well-designed studies aimed at clarifying the impact of the type of feeding, as well as related confounding factors, on the relationship between probiotic supplementation and clinical outcome. Furthermore, future research is needed to provide solid evidence about the potential greater efficacy of multi-strain/multi-species probiotics compared to single-strain products. According to recent observations, paraprobiotics could represent an answer to safety concerns related to the routine administration of probiotics in preterm infants, and pre-clinical and clinical studies should be targeted to explore this new frontier. Further RCTs exploring probiotic supplementation in preterm infants should also consider a cluster design to avoid the issue of cross-contamination. Last, in light of increasing commercial and public interests, the long-term effect of the routine consumption of probiotics and the risk connected to antibiotic resistance in this vulnerable population should be explored in ad hoc studies.
